# Marine-Derived Anticancer Agents: Clinical Benefits, Innovative Mechanisms, and New Targets

**DOI:** 10.3390/md17060329

**Published:** 2019-06-02

**Authors:** Renato B. Pereira, Nikolai M. Evdokimov, Florence Lefranc, Patrícia Valentão, Alexander Kornienko, David M. Pereira, Paula B. Andrade, Nelson G. M. Gomes

**Affiliations:** 1 REQUIMTE/LAQV, Laboratório de Farmacognosia, Departamento de Química, Faculdade de Farmácia, Universidade do Porto, R. Jorge Viterbo Ferreira, n° 228, 4050-313 Porto, Portugal; ren.pereira@gmail.com (R.B.P.); valentao@ff.up.pt (P.V.); dpereira@ff.up.pt (D.M.P.); 2 Department of Chemistry and Biochemistry, University of California, Santa Barbara, CA 93106, USA; nevdokim@chem.ucla.edu; 3 Department of Neurosurgery, Hôpital Erasme, Université Libre de Bruxelles, 808 Route de Lennik, 1070 Brussels, Belgium; florence.lefranc@erasme.ulb.ac.be; 4 Department of Chemistry and Biochemistry, Texas State University, San Marcos, TX 78666, USA; a_k76@txstate.edu

**Keywords:** cytarabine, trabectedin, eribulin, brentuximab vedotin, plitidepsin, lurbinectedin, plinabulin, marizomib, plocabulin, antibody–drug conjugates

## Abstract

The role of the marine environment in the development of anticancer drugs has been widely reviewed, particularly in recent years. However, the innovation in terms of clinical benefits has not been duly emphasized, although there are important breakthroughs associated with the use of marine-derived anticancer agents that have altered the current paradigm in chemotherapy. In addition, the discovery and development of marine drugs has been extremely rewarding with significant scientific gains, such as the discovery of new anticancer mechanisms of action as well as novel molecular targets. Approximately 50 years since the approval of cytarabine, the marine-derived anticancer pharmaceutical pipeline includes four approved drugs and eighteen agents in clinical trials, six of which are in late development. Thus, the dynamic pharmaceutical pipeline consisting of approved and developmental marine-derived anticancer agents offers new hopes and new tools in the treatment of patients afflicted with previously intractable types of cancer.

## 1. An Overview on Fifty Years of Marine-Derived Drug Discovery

**Figure 1:**
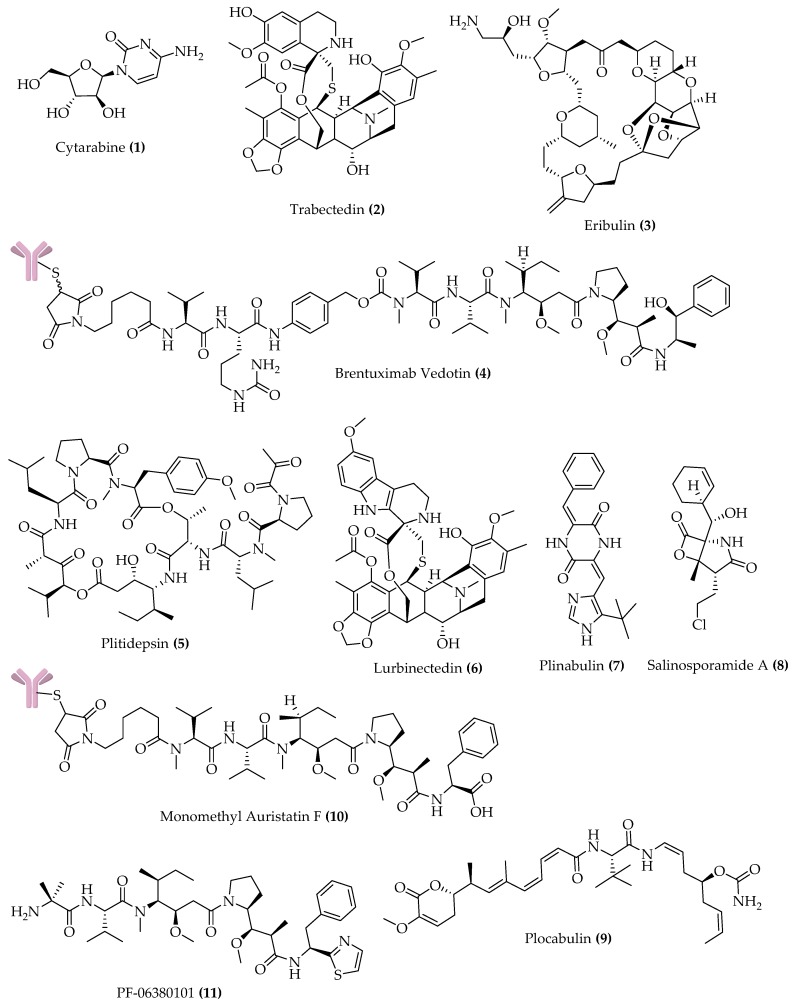
Structures of marine-derived licensed drugs and clinical candidates.

Soon after the landmark discovery of the DNA double helix structure, the isolation of two arabinose-containing nucleosides from the sponge *Cryptotethya crypta* [1] was the first step for the development of cytarabine (**1**) (Figure 1), the first approved marine-derived drug [2]. Immune to the new paradigm set by the emergence of combinatorial chemistry and the advent of high throughput screening in the 1990s [3], natural product researchers switched from the terrestrial environment to the oceans, with more than 28,600 marine natural products reported as a result of their efforts [4]. With over 50% of the new bioactive marine natural products isolated during the period of 1985–2012 exhibiting cytotoxicity toward experimental models of cancer [5], marine bioprospection has been particularly rewarding in the area of cancer, with four chemotherapeutic agents already approved and eighteen additional drug candidates enriching the oncological pipeline (Table 1). The development of these anticancer drugs corroborated the unmeasurable impact of natural products on the current chemotherapeutic armamentarium, with 49% of anticancer agents approved prior to 2014 being classified either as natural products or directly derived therefrom [6]. In fact, the chemical diversity of drugs compared to natural products and synthetic libraries has shown that the chemical diversity of natural products is more closely aligned with drugs than synthetic libraries [7], in contrast with the common assumption that most drugs have a purely synthetic origin [6]. Despite the inherent limitations associated with the drug discovery and development from marine sources, the progress in analytical instrumentation [8], anticancer screening platforms [9], scalable synthetic approaches [10], and antibody–drug conjugates (ADCs) [11] allowed the broadening of the clinical arsenal for cancer treatment. In addition to the evident clinical benefits, additional scientific gains have been witnessed with the development of these innovative anticancer agents.

## 2. Marine Chemotherapeutic Pipeline and Clinical Benefits

### 2.1. Licensed Drugs

Nearly 50 years after the approval of cytarabine, the contribution of the marine ecosystem on the development of anticancer drugs appears to be modest, a biased overlook easily refuted due to the undeniable clinical utility of the drugs licensed so far.

Following the studies with animal models [12], several research groups, namely the Acute Leukemia Group B and the Children’s Cancer Study Group A, tested the antimetabolite cytarabine in patients with different phenotypes of acute myelogenous leukemia (AML). The results strengthened cytarabine’s usefulness as a cancer drug, leading to superior remissions at all age levels compared to other drugs available in the 1960s, revolutionizing the treatment and management of hematological malignancies [13,14]. Cytarabine was licensed by the Food and Drug Administration (FDA) under the tradename Cytosar-U^®^ for the treatment of AML, remaining as a mainstay drug in the management of AML, as well as in the treatment of acute lymphoblastic leukemia (ALL) and chronic myelogenous leukemia (CML) [2]. Mainly used in polychemotherapy, cytarabine formed the backbone of the most frequently adopted regimens, namely the salvage therapy MEC (mitoxantrone, etoposide and cytarabine), DHAP (dexamethasone, cytarabine and cisplatin), and the ESHAP (etoposide, methylprednisolone, high dose cytarabine and cisplatin) regimens [2], as well as the “7 + 3 regimen” established in 1973 that formed the backbone of induction and consolidation therapy for most patients with AML for decades [15]. Years later, a slow-release liposomal formulation was developed, displaying significant advantage over the cytarabine standard formulation and other conventional drugs, allowing a longer progression-free survival (PFS) and a convenient dosing schedule [16,17]. Results from a randomized, multicenter trial demonstrated a remarkably higher response rate (71%) compared to that produced by free cytarabine (15%) in patients with lymphomatous meningitis [16], with further trials providing evidence of DepoCyt^®^ safety and efficacy in the treatment of solid tumor neoplastic meningitis [16,17,18]. DepoCyt^®^ received accelerated approval by the FDA in 1999 and marketing authorization by the European Medicines Agency (EMA) in 2001 for the intrathecal treatment of lymphomatous meningitis, with it also being used for the treatment of leptomeningeal metastasis in solid tumors [19].

Nearly five decades after cytarabine’s approval, over 1000 clinical trials are listed in European and US databases, with more than 150 studies in phase 3 [20,21].

Originally reported from the Caribbean tunicate *Ecteinascidia turbinata* [22], trabectedin (**2**) (Figure 1) (Yondelis^®^) was granted an accelerated approval by EMA in 2007 as a single agent for the treatment of advanced soft tissue sarcoma in adults after failure of anthracyclines and ifosfamide, or as a first line therapy in patients who cannot receive these agents [23]. Trabectedin was only approved by the FDA in 2015, based on the results of a phase 3 trial demonstrating a significant improvement in PFS compared to dacarbazine in patients with metastatic liposarcoma or leiomyosarcoma [23]. Preliminary results of a worldwide expanded access program, including more than 1800 patients with liposarcoma and leiomyosarcoma, confirmed trabectedin’s clinical benefit, particularly in certain histological subtypes, leading to significantly longer overall survival (OS) mainly in the myxoid/round-cell liposarcoma variant [24]. Based on the results from the phase 3 trial OVA-301, the combination of trabectedin and pegylated liposomal doxorubicin (PLD) improved PFS and overall response rate (ORR) over PLD alone [25,26]; trabectedin received a second marketing authorization by EMA in 2009. Following the initial EMA approval, patients with relapsed platinum-sensitive ovarian cancer benefited from trabectedin in combination with PLD for second line therapy in more than 65 countries worldwide [27]. The feedback from ten years of use has shown an acceptable toxicity profile, without evidence of cumulative side effects. However, due to an extensive hepatic metabolization, liver dysfunction, predominantly characterized by increased transaminase levels, was reported as a common side effect [23]. Despite the tendentiously transitive and non-cumulative transaminitis, co-medication with glucocorticoids is mandatory and has been proven to reduce both hepatotoxicity and myelosuppression [28]. Relevantly, unlike doxorubicin, trabectedin treatment is not associated with cumulative cardiotoxicity, demonstrating also a more favorable safety profile in comparison with ifosfamide [29].

Based on halichondrin B, a complex macrolide isolated from the rare sponge *Halichondria okadai* [30], the simplified synthetic analog eribulin (**3**) (Figure 1), commercialized under the tradename Halaven^®^, was the third anticancer agent to receive market authorization. Based largely on the favorable results from the phase 3 trial EMBRACE [31], eribulin met approval by the FDA and EMA as a monotherapy for the treatment of patients with metastatic breast cancer who previously received an anthracycline and a taxane in either the adjuvant or metastatic setting and at least two chemotherapeutic regimens for the treatment of metastatic disease [32]. So far approved in more than 50 countries, eribulin is the only anticancer drug in the last decade showing increased OS, with an acceptable toxicity profile in heavily pre-treated patients with metastatic breast cancer, refractory to other microtubule-targeting agents [33]. Relevantly, despite the reports on its in vitro metabolization by CYP3A4, no in vivo interactions have been documented after combined administration with CYP3A4 modulators [34]. Consequently, a minimal risk was observed of clinically relevant interactions with anticancer agents metabolized by CYP3A4, such as tamoxifen and paclitaxel, unless they are concomitantly potent P-glycoprotein (P-gp) inhibitors [34,35]. This therapy has set a new paradigm in breast cancer treatment, predominantly targeting hormonal receptors through HER2 receptor-drugs or aromatase inhibitors, with more than limited efficacy in triple-negative breast cancer [36]. Relevantly, the phase 1b/2 study ENHANCE-1, which evaluated the combination of eribulin and pembrolizumab in patients with metastatic triple-negative breast cancer, demonstrated a 33.3% objective response rate, the primary efficacy endpoint of the study [37]. Furthermore, a unique characteristic of eribulin deals with a rapid intravenous administration over 2–5 min, in contrast with other antitubulin agents which require long infusion times [31]. Another marketing authorization for the treatment of patients with unresectable or metastatic liposarcoma who received a prior anthracycline-containing regimen was issued in 2016 by the FDA. The EMA Committee for Medicinal Products for Human Use (CHMP) also adopted a positive opinion, recommending a new marketing authorization in the same clinical setting. While there was no relevant evidence of clinical efficacy, eribulin demonstrated a significant improvement in OS, making it the first agent to gain approval based on survival for patients with liposarcoma [38].

The discovery of the pentapeptide dolastatin 10 from the sea hare *Dolabella auricularia* set the cornerstone for the development of the CD30-targeted ADC brentuximab vedotin (Adcetris^®^) (**4**) (Figure 1) [39]. Brentuximab vedotin consists of a chimeric IgG1 monoclonal anti-CD30 antibody covalently linked, via a protease-cleavable dipeptide linker, to the dolastatin 10 synthetic analog monomethyl auristatin E (MMAE) [39,40]. The efficacy and safety as a single agent were demonstrated in two pivotal phase 2 studies; brentuximab vedotin led to an objective response rate of 75% in patients with histologically documented CD30^+^ relapsed or refractory Hodgkin lymphoma, leading to complete remission in 33% of the patients and tumor reductions detected in 94% [41]. In relapsed or refractory systemic anaplastic large-cell lymphoma (sALCL), the drug mediated 86% of ORRs, with 97% of tumors being reduced in size and over 50% patients achieving complete remission [42]. On the basis of the compelling objective responses observed in the two trials, brentuximab vedotin received accelerated approval by the FDA in 2011 for the treatment of Hodgkin lymphoma patients that relapsed after autologous stem cell transplantation (ASCT), or after at least two prior lines of multidrug regimens, with ineligibility for ASCT in the second line setting and for the treatment of systemic anaplastic large-cell lymphoma (sALCL) after failure of at least one multi-agent chemotherapy regimen [43]. A conditional marketing authorization was granted by the EMA in 2012 for the restricted treatment of adult patients with relapsed or refractory CD30^+^ Hodgkin lymphoma after ASCT or after at least two previous therapies when ASCT or multiagent therapy is not a treatment option and for the treatment of adult patients with relapsed or refractory sALCL [44]. Long term follow-up data showed that several patients with Hodgkin lymphoma remained in remission after more than four years, which is suggestive of prolonged disease control [45,46]; PFS of more than 14 months was also reported in the sALCL clinical setting [47]. Reported drug interactions with brentuximab vedotin have been attributed to the monomethyl auristatin E (MMAE) portion, which is mainly metabolized via CYP3A4/5 and requires close monitoring in patients receiving strong CYP3A4 modulators that may influence the systemic exposure of the warhead [48]. Despite the improved efficacy and manageable toxicity in the concomitant treatment with other anticancer agents [49], co-administration with bleomycin is contraindicated due to excessive pulmonary toxicity [50], as well as with vinca-domain-interacting drugs due to an increased risk of cumulative neurotoxicity [51].

Adcetris^®^ is the first anticancer drug that selectively targets malignant Hodgkin and Reed–Sternberg cells, being concomitantly an innovative approach by influencing the tumor microenvironment [52]. In the last 50 years, the approved agents for relapsed T-cell lymphomas are mostly all-comers not selected based on the subtype. Adcetris^®^ has offered newly diagnosed patients the frontline treatment since the establishment of the ABVD (doxorubicin, bleomycin, vinblastine and dacarbazine) regimen in 1997 [52].

### 2.2. Agents in Clinical Trials

An overview on the dynamic oncological pipeline based on marine-derived metabolites, currently including 18 candidates in clinical development, suggests that the range of chemotherapy agents will soon be enriched. Particularly considering the six late-stage drug candidates, results from clinical trials demonstrate preliminary but evident clinical benefits.

Displaying a unique cytotoxic fingerprint and identified as a US-NCI-COMPARE-negative compound, plitidepsin (Aplidin^®^) (**5**) (Figure 1), originally isolated from the Mediterranean tunicate *Aplidium albicans*, rapidly proceeded to clinical development [53,54]. While several phase 2 studies revealed limited clinical activity in solid tumors, plitidepsin led to objective benefits in hematological cancers [55]. Clinical benefits were particularly evident in multiple myeloma, with results from a phase 2 trial in a relapsed or refractory setting demonstrating a response rate of 13% as a single agent and 22% in co-administration with dexamethasone [56]. The phase 3 trial ADMYRE, concluded in November 2017, showed that combinatorial therapy led to prolongation of both PFS and OS in patients with relapsed/refractory multiple myeloma who failed conventional therapy, with a remarkable duration of response, which also reassured the plitidepsin safety profile [57]. Manageable and reversible dose-limiting toxicities were mainly characterized by muscular and hepatic adverse effects [58,59]. Myotoxicity was characterized by increased serum creatine phosphokinase (CPK) levels, correlated with myalgia and muscle weakness, but was prevented through the co-administration of L-carnitine [59]. Despite the negative opinion adopted by the EMA-CHMP, Aplidin^®^ received orphan drug status for the treatment of multiple myeloma in Switzerland in 2017, following the recognition granted by the FDA in 2004 [60].

Differing from trabectedin through a minor structural variation, the synthetic derivative lurbinectedin (Zepsyre^®^) (**6**) (Figure 1) is another late-stage candidate developed by PharmaMar SA. The minimal structural dissimilarities were found to result in unexpected pharmacokinetic and pharmacodynamic advantages in comparison with its natural counterpart, as evidenced in a phase 1 study in patients with advanced solid tumors which established a maximum tolerated dose of 5.0 mg/m^2^ for lurbinectedin, a remarkably higher dose than trabectedin (1.5 mg/m^2^) [61]. In a phase 2 trial, lurbinectedin’s significant superiority over topotecan was demonstrated in terms of ORR, PFS, and OS in patients with platinum-resistant/refractory ovarian cancer [62]. However, lurbinectedin failed the primary endpoint of PFS in comparison with standard chemotherapy in the phase 3 study CORAIL [63], despite a better safety profile claimed by PharmaMar SA. An active phase 3 global registration trial (ATLANTIS) is currently underway which is investigating the difference in PFS between lurbinectedin/doxorubicin *versus* cyclophosphamide, doxorubicin, and vincristine or topotecan in small-cell lung cancer (SCLC) after platinum therapy. Preliminary data presented at the American Society of Clinical Oncology’s (ASCO’s) annual meeting, showed an OS benefit of 11.8 months [64].

As noted, most marine-derived anticancer drugs and candidates refer to metabolites originally reported from invertebrates or their synthetic derivatives. Strictly referring to marine fungal agents, plinabulin (**7**) (Figure 1), a microtubule-disrupting agent based on the fungal agent halimide [65] is the only candidate that has undergone clinical development to date. Based on the encouraging results obtained in preclinical models, plinabulin was brought into clinical trials for the treatment of non-small-cell lung cancer (NSCLC) [66]. Clinical studies to date demonstrate that plinabulin protects against the development of chemotherapy-induced neutropenia (CIN), which appears to be related to its capacity to facilitate the release of cytokines, protecting neutrophils from apoptosis [67]. On the other hand, due to structural and pain mediator effects on surrounding tissues from the tumor necrosis, tumor pain is a known consequence of plinabulin and other vascular-disrupting agents’ usage, being generally managed with analgesics and/or improved with continued treatment [68]. The addition of plinabulin to docetaxel in NSCLC patients with a measurable lesion led to encouraging results, improving the median OS by 4.6 months when compared to docetaxel alone [69]. Based on these findings, a global phase 3 trial (DUBLIN-3) was initiated to evaluate second- or third-line treatment with docetaxel plus plinabulin in patients with advanced NSCLC with at least one measurable lung lesion. Moreover, a phase 2/3 trial (Protective-1) is evaluating the duration of severe neutropenia with plinabulin *versus* pegfilgrastimin in patients with solid tumors receiving docetaxel myelosuppressive chemotherapy [70]. Additional phase 3 trials are still recruiting patients to evaluate the combination of plinabulin and nivolumab in NSCLC [71].

In the same year that the proteasome was validated as an oncological drug target through the FDA approval of bortezomib for the treatment of patients with multiple myeloma, the irreversible proteasome inhibitor salinosporamide A (NPI-0052, marizomib) (**8**) (Figure 1) was discovered during the screening of extracts from the marine actinomycete *Salinispora tropica* [72]. Extensive and promising preclinical data suggested that the unusual structure of marizomib produces unique signal transduction, safety, and efficacy profiles compared with other proteasome inhibitors, breaking ground to the clinical trials [73]. Results from a phase 1 trial (NPI-0052-102) in patients with advanced malignancies demonstrated activity in heavily pre-treated relapsed/refractory multiple myeloma patients, with good safety and a non-cross-reactive toxicity profile and not leading to myelosuppression, peripheral neuropathy, or thrombocytopenia, which are observed with other proteasome inhibitors [74]. Marizomib has been evaluated in more than 230 patients across four phase 1/2 studies, either as a single agent or in combination with dexamethasone or a histone deacetylase (HDAC) inhibitor, providing superior potency, specificity, and duration of proteasome inhibition and potentially improved clinical activity, with it being termed as orphan drug against multiple myeloma by the FDA and EMA [75]. Based on encouraging dose-escalation results from a phase 1/2 trial in combination with temozolomide and radiotherapy in newly diagnosed glioblastoma [76], marizomib progressed to late-stage clinical development in the same clinical setting, receiving orphan drug designation for glioblastoma in the United States.

Originally described in the Madagascan sponge *Lithoplocamia lithistoides* by PharmaMar SA researchers, the polyketide plocabulin (PM060184, PM184) (**9**) (Figure 1) displays the highest known affinities among tubulin-binding agents [77,78]. Based on its sub-nanomolar potency and a distinct inhibition mechanism on microtubules in addition to the results from in vivo studies [78], plocabulin entered clinical development, with it presently being evaluated in phase 1/2 trials in patients with advanced malignancies [79].

Notably, 13 of the marine-derived anticancer candidates correspond to ADCs and are therapies that directly target tumor cells through the conjugation of monoclonal antibodies with the auristatin class of microtubule-disrupting agents [80]. The successful development of Adcetris^®^ sparked the design of additional candidates sharing the cysteine-linker-auristatin motif.

Polatuzumab vedotin (DCDS4501A) and pinatuzumab vedotin (DCDT2980S), MMAE-based ADCs targeting CD79b and CD22, respectively, showed clinical activity in combination with rituximab in relapsed/refractory diffuse large B-cell lymphoma (DLBCL) [81]. Due to the greater clinical efficacy of polatuzumab vedotin, the phase 3 trial POLARIX is underway, comparing the efficacy and safety in combination with R-CHP (rituximab, cyclophosphamide, doxorubicin and prednisone) and R-CHOP (rituximab, cyclophosphamide, doxorubicin, vincristine and prednisolone) regimens in the same clinical setting [82,83]. In 2017, polatuzumab vedotin was granted *Breakthrough Therapy* designation by the FDA, as well as PRIME designation by the EMA [84]. Also, the late-development candidate enfortumab vedotin (ASG-22ME) was granted *Breakthrough Therapy* designation by the FDA for patients with locally advanced or metastatic urothelial cancer who were previously treated with immune checkpoint inhibitors [85].

ADCs made with valine-citrulline MMAE (vcMMAE) display a similar toxicity profile, characterized by acute neutropenia and neuropathy as the dose-limiting adverse events, irrespective of the target antigen [86]. Nevertheless, certain toxicities observed in clinical trials appear to be on-target effects, as in the case of glembatumumab vedotin (CDX-011). The development of a skin rash is one of the observed dose-limiting toxicities, apparently due to membrane expression of glycoprotein non-metastatic b (gpNMB) in epithelial cells of the skin [87]. Despite the recent failure in triple-negative breast cancer (METRIC phase 2 trial), glembatumumab vedotin continues clinical evaluation in a number of early-phase clinical trials in patients with osteosarcoma, uveal melanoma, or lung squamous cell carcinoma overexpressing gpNMB [88]. In addition to the abovementioned candidates, six additional MMAE-coupled ADCs are presently registered in US and European databases in early-stage trials.

The recent development of new linkers and cytotoxic payloads with improved potency has been successfully translated to the design of additional ADCs. Listed in clinical trials databases, ABT-414 [89], AGS-16C3F [90], and GSK2857916 [91] comprise a distinct ADC architecture using monomethyl auristatin F (MMAF) (**10**) (Figure 1) as a warhead, conjugated with an antibody via a plasma-stable maleimido-caproyl linker. Relevantly, this new linker-payload format minimizes off-target effects derived from MMAF low cell permeability, thus preventing the diffusion across membranes and reducing the potential toxicity for the surrounding cells [92]. While this effect derives from a limited bystander effect, it may avoid the potential for neurotoxicity in particular clinical settings such as glioblastoma [89]. In fact, results from the INTELLANCE 2 trial with the late-stage candidate depatuxizumab mafodotin (ABT-414; Deputax-M) in combination with temozolomide showed OS improvement in epidermal growth factor receptor (EGFR)-amplified recurrent glioblastoma [93]. Based on a pediatric sub-study nested with INTELLANCE 2, the FDA granted rare pediatric disease designation to the drug for the treatment of children with EGFR-amplified diffuse intrinsic pontine glioma (DIPG) [94]. Two phase 3 trials are currently underway, testing depatuxizumab mafodotin in subjects with newly diagnosed glioblastoma with EGFR amplification (Intellance 1) as well as the evaluation of ophthalmologic prophylactic treatment strategies for the management of ocular side effects in participants being treated with the drug [95,96].

Later, the synthesis of the new lead auristatin PF-06380101 (**11**) (Figure 1), which displays excellent potency in tumor proliferation assays and a differential ADME profile, allowed the development of PF-06647020 and its recent progression to clinical evaluation against advanced solid tumors, sponsored by Pfizer [97].

## 3. New Routes in Oncological Research—Innovative Mechanisms and New Molecular Targets

As noted above, the discovery and development of marine-derived anticancer drugs has broadened the scope of action in the treatment of cancer. Relevantly, the progress achieved is not only characterized by direct clinical benefits but also by the discovery of new mechanisms of action and molecular targets as associated scientific outcomes, establishing new avenues for the development of alternative chemotherapeutic agents as well as the understanding of the events associated with carcinogenesis and subsequent progression of the disease.

The structurally-related minor-groove alkylators trabectedin and lurbinectedin share a complex pleiotropic anticancer mechanism, affecting not only the tumor cells, but also the tumor microenvironment. Differing from conventional alkylators, both agents bind to the exocyclic amino group of guanines, with preference for guanine-cytosine-rich triplets of the DNA minor groove, causing an atypical bending toward the major groove [98,99]. Consequently, a cascade of synergistic events is catalyzed, leading to an arrest of proliferation, differentiation, and cell death [100]. In addition to the binding moiety (subunits A and B), another subunit (subunit C) protrudes from the DNA backbone, interacting directly and indirectly with different DNA-binding proteins, such as transcription factors or DNA repair proteins [101,102]. Experimental and clinical studies revealed the particular efficacy of trabectedin against soft-tissue sarcomas, derived from its unique molecular feature not shared by other anticancer drugs [29,103]. The unparalleled sensitivity of translocation-related sarcomas is apparently associated with the displacement of oncogenic fusion proteins, which is critical for neoplastic transformation and tumor progression [104]. Trabectedin is particularly effective in myxoid liposarcomas, displacing the fusion protein FUS-CHOP from its target DNA promoters and impairing the trans-activating capacity of the FUS-CHOP chimaeras, leading to a reversal effect of the oncogenic program and phenotype in myxoid liposarcoma cells, restoring their ability to drive adipocyte differentiation [104,105]. The atypical pattern of sensitivity was also observed in cells with differential proficiency of DNA repair mechanisms, namely the transcription-coupled nucleotide excision repair (TC-NER) and homologous recombination (HR) systems. In contrast to other DNA-binding agents, cells with functional impairment of NER were significantly resistant to trabectedin and lurbinectedin [106,107], while HR-deficient cells displayed increased sensitivity [106,108]. By trapping xeroderma pigmentosum complementation group G (XPG)-DNA complexes, trabectedin blocks TC-NER and prevents further processing, thus causing DNA single-strand breaks [101,109]. The high sensitivity of cells that are deficient in HR repair systems evokes a particular effect toward breast and ovarian tumors with BRCA1 or BRCA2 mutations [110,111]. Relevantly, in vitro studies also revealed a potential enhancement of cytotoxicity in cancer cells resistant to doxorubicin and vincristine derived from trabectedin’s ability to downregulate P-gp, which showed its usefulness in combination with other chemotherapeutic agents that are P-gp substrates [112]. While the direct and indirect effects on the neoplastic cell compartment seem to be the main mechanisms behind the anticancer effects, trabectedin and lurbinectedin also target the tumor microenvironment, apparently contributing to a delayed response with prolonged stabilization of disease [113]. Besides a direct cytotoxic effect on mononuclear phagocytes, both agents influence the communication of inflammatory and cancer cells. This communication is also prevented through the downregulation of the expression of inflammatory mediators, limiting the recruitment of circulating monocytes and thus depriving the tumor of the inflammatory-mediated support [114,115,116]. The pronounced anticancer efficacy is also associated with an evident regression of capillary networks related to the reduced production of the inflammatory chemokines CCL2, CXCL8, and IL-6 as well as angiogenic factors [105,115,117].

Scientific spin-offs associated with the development of marine-inspired drugs also include the discovery of agents with unique effects on tubulin as well as a new pharmacophore. As the natural counterpart halichondrin B, eribulin shares a similar antiproliferative pattern with other antitubulin agents but exhibits distinct effects on microtubule dynamics, apparently involving the binding to either the interdimer interface or the β-tubulin subunit alone, acting via an end-poisoning mechanism [118,119]. Sub- to low-nanomolar levels of eribulin inhibit microtubule polymerization by binding predominantly to a small number of high-affinity sites on the growing plus ends of microtubule protofilaments, rather than along their lengths, with no effect on microtubule shortening, unlike taxanes, vinca alkaloids, and epothilones [119,120]. In addition to the action on tubulin polymerization, eribulin induces the sequestration of tubulin into non-productive globular aggregates, leading to the disruption of the mitotic spindle, thus blocking the cell cycle progression at the metaphase/anaphase checkpoint [118]. The unique effects of eribulin on microtubule dynamics may explain its clinical efficacy in taxane-refractory disease and the ability to circumvent taxane resistance, particularly in breast cancer with high levels of βIII-tubulin isotype expression, correlated with the resistance to conventional tubulin-based antimitotic agents [120]. A renewed interested in eribulin was witnessed due to additional preclinical results, showing a novel anti-mesenchymal mechanism of action characterized by phenotype reversal from epithelial–mesenchymal transition (EMT) to mesenchymal–epithelial transition (MET) states [121]. Notably, a potent antiproliferative effect was also observed in human umbilical endothelial cells, displaying an activity comparable to that of paclitaxel as well as decreased expression of the angiogenesis-related genes delta-like ligand 4 (DLL4) and platelet-derived growth factor receptor β (PDGFR*β*) [122]. By interfering with tumor vasculature remodeling, resulting in the reduction of the tumor microenvironment abnormality associated with drug resistance and metastasis, eribulin clinical efficacy may also be partially dependent on those effects [122]. It was hypothesized that the weakened aggressiveness of tumors, together with the reduction of inner tumor hypoxia, could partially contribute to eribulin’s anticancer effects against tumors that are resistant to other antimicrotubule drugs, such as taxanes and vinca alkaloids [122].

Unlike eribulin, plocabulin suppresses microtubule shortening and growing to a similar extent, interfering with the dynamic instability of microtubules and affecting cells, both in interphase and mitosis [123]. Strong evidence suggests that these effects are derived from a new form of interaction with the tubulin dimer, sharing a common tubulin-binding site with rhizoxin and concomitantly interfering with the binding of vinblastine [77,78]. The unparalleled binding mode on tubulin was preliminarily suggested in PM060184-resistant mutants of *Aspergillus nidulans*, which involves the interaction with a new locus in β-tubulin defined by the position Asn100. This was later confirmed by X-ray crystallography, corroborating the discovery of the maytansine binding site as a new pharmacophore [124]. In addition to the effects on the microtubule network, the antitumor activity of PM060184 seems also to depend on relevant anti-angiogenic properties, namely the inhibition of migration and invasion described in human umbilical vein endothelial (HUVEC) cells [79].

The discovery of the microbial metabolite marizomib set a new paradigm with the development of second-generation proteasome inhibitors, due to its distinct activity and specificity. In contrast with other proteasome inhibitors such as bortezomib and carfilzomib, marizomib rapidly enters cells and irreversibly binds to all three active enzyme sites in the 20S proteasome, termed caspase-like (C-L, β1), trypsin-like (T-L, β2), and chymotrypsin-like (CT-L, β5) subunits [125,126]. The complex and densely functionalized γ-lactam-β-lactone bicyclic core seems to be preponderant to its distinct mode of action, requiring cell replacement and/or proteasome re-synthesis to revert its effect [127,128]. Several studies indicate that overexpression of the β5 proteasome subunit is the primary response mechanism to proteasome inhibition, which may precede acquisition of β5 mutations as well as increased β1 and β2 activity following prolonged bortezomib exposure [129,130]. Once marizomib binds irreversibly to all three proteasome subunits, it maintains a long-term inhibition, also overcoming the compensatory hyperactivation of C-L and T-L subunits in response to the CT-L blockade, which is partially responsible for the chemoresistance observed with reversible proteasome inhibitors [128]. Moreover, studies carried out in several tumor xenograft models demonstrated that marizomib displayed superior activity than bortezomib, potently affecting several hallmarks of cancer including angiogenesis and invasion [131].

The application of the Trojan horse concept to the auristatin class of cytotoxic agents and the subsequent development of ADCs to target a wide range of antigens was also accompanied by remarkable achievements in the validation of molecular targets. The development of pinatuzumab vedotin allowed the further substantiation of CD22 as a clinically validated target, paving the way for the development of novel ADCs based on distinct cytotoxic agents as warheads targeting CD22 [132]. In vivo studies in xenograft models exposed to the MMAE-based ADC tisotumab vedotin (HuMax^®^-TF-ADC), the first ADC employing a tissue factor-specific antibody to deliver a cytotoxic agent [133], demonstrated that the new ADC outperformed HER2-ADC and EGFR-ADC in two different tumor xenograft models, indicating that tissue factor is a highly suitable target for the intracellular delivery of cytotoxic agents through an ADC [134]. Relevantly, the development of ladiratuzumab vedotin (SGN-LIV1A), AGS67E, and ASG15ME, also employing MMAE as a payload, allowed the identification of specific genes in cancers with distinct etiologies. Preclinical testing of ladiratuzumab vedotin revealed a pronounced activity as a single agent in preclinical models of breast cancer, showing also that LIV-1 is expressed in all subtypes of breast cancer, including triple-negative [135]. In addition to the potent anticancer efficacy in preclinical patient-derived models of AML upon treatment with AGS67E, it was reported for the first time that CD37 is highly expressed in T-cell lymphomas and AML, thus allowing the exploration of the possibility of using the antigen as a target in these clinical settings [136]. Finally, the development of ASG15ME has shown for the first time that SLITRK6 is well-expressed in non-invasive and invasive bladder cancer [137].

## 4. Additional Technological Improvements

Despite the clinical benefits derived from the access to new antitumor agents of marine origin and the promising results obtained with various investigational candidates, the adjacent technological progress associated with their development has been notable. The limitations inherent to the supply and production of these revolutionizing anticancer agents at laboratory and industrial scales have been surpassed with the development of fermentative processes and scalable synthetic methods.

While some of these agents are naturally-occurring, the production of the agents currently constituting the marine-derived oncological pipeline has been mainly ensured through their chemical synthesis. A notable exception refers to marizomib manufacturing, with original fermentation conditions for laboratory scale production yielding only a few milligrams per liter [138]. In order to industrialize and deliver marizomib at a suitable scale and quality for human administration, Nereus developed a robust saline fermentation process, employing a wild type strain obtained from *Salinispora tropica*, corresponding to the first manufacture of clinical trial materials by saline fermentation under cGMP [138].

Synthesis has played a pivotal role in the provision of marine metabolites for clinical trials and production of approved pharmacological agents. A case in point is the development of a scalable synthetic route to trabectedin, an approved anticancer agent as discussed earlier in this review. The original temporary solution to the supply problem was provided by a total synthesis of this natural product by Corey’s group [139]. The synthesis involved 46 steps and generated the final product in 1% overall yield. Subsequently, it was improved by a semisynthetic approach developed by scientists at PharmaMar, which is currently utilized for its industrial preparation for clinical use [140]. It is important, however, to appreciate the discovery of a large number of synthetic methods during these investigations. Thus, Corey’s route involved the discovery of novel variations of Pictet–Spengler cyclization, which is important for the synthesis of heterocyclic structures. The synthesis also led to the development of innovative transannular annulations and quinone methide addition chemistry. The semisynthesis of trabectedin by PharmaMar led to the successful demonstration of Edman degradation and Rapoport deamination in the context of complex natural product structures. All these discoveries represent important contributions to basic science of chemical synthesis that can be used by synthetic chemists in the future.

## 5. Conclusions

Nearly 70 years after the discovery of the spongonucleosides spongouridine and spongothymidine, the current chemotherapeutic clinical pipeline is enriched with 18 candidates at different stages of development, in addition to four anticancer agents that have revolutionized the current paradigm in oncological therapy. The potential of marine organisms as prolific producers of compounds that may be useful as inspiring tools to develop new anticancer agents may be questioned due to an apparent limited number of candidates. However, several aspects related to the specificities of marine-sourced drug discovery and development should be rationalized and taken into account. In contrast to the ethnopharmacological approach that led to the identification of several metabolites produced by plants, which ultimately allowed the development an impressive therapeutic arsenal, it is conceivable to consider that the bioprospection of the marine environment, with the discovery of new pharmacologically useful agents, started in the late 1980s. Since then, an exciting exponential growth on the discovery of new chemical entities attracted a considerable curiosity, firstly from natural product chemists and later from clinicians. Despite the early preclinical studies reporting outstanding pharmacological properties of several marine-derived compounds, it was later shown that particular limitations would be associated with their development and, ultimately, with their availability as therapeutic tools. The efforts on overcoming these inherent limitations led to remarkable achievements, such as the provision of salinosporamide A, through the first industrial-scale saline fermentation process to meet cGMP guidelines and the semisynthetic approach for the provision of trabectedin, starting from the bacterial intermediate cyanosafracin B.

While the development of cost-effective methods to ensure an appropriate supply have proved that these advances were not a merely academic exercise, the clinical development of some of these marine-derived agents also led to other outcomes in addition to the clinical benefit. The discovery of prototype structures with remarkable potencies, ranging from picomolar to nanomolar, is outstanding per se; however, the progress on the identification of new molecular targets and new chemotherapeutic approaches also provide unmeasurable scientific benefits. While the current review focuses on marine-derived anticancer agents already approved or in clinical development, it is expected that the increasing number of early candidates in preclinical investigations will not only be translated in new chemotherapeutic tools, but also in the discovery of additional molecular targets.

## Figures and Tables

**Table 1:** Marine-derived chemotherapeutic pipeline. ^1.^

Compound Name (Trademark)	Lead Compound (Source)	Chemical Class	Molecular Target	Cancer Conditions
**APPROVED**				
**Cytarabine** **(Cytosar-U^®^; DepoCyt^®^)**	Spongothymidine (Sponge)	Nucleoside	DNA polymerase	Leukemia; lymphomatous meningitis
**Trabectedin** **(Yondelis^®^)**	Trabectedin (Tunicate)	Alkaloid	DNA minor groove	Soft tissue sarcoma; ovarian cancer
**Eribulin mesylate** **(Halaven^®^)**	Halichondrin B (Sponge)	Macrolide	Microtubules	Metastatic breast cancer; advanced liposarcoma
**Brentuximab vedotin** **(Adcetris^®^)**	Dolastatin 10 (Mollusk/cyanobacterium)	ADC ^a^ (MMAE) ^b^	CD30 and microtubules	sALCL ^c^; Hodgkin lymphoma
**PHASE 3**				
**Plitidepsin** **(Aplidin^®^)**	Plitidepsin (Tunicate)	Depsipeptide	Rac1 and JNK activation	Relapsed/refractory multiple myeloma
**Lurbinectedin** **(Zepsyre^®^)**	Trabectedin	Alkaloid	DNA minor groove	Ovarian cancer; SCLC ^d^
**Plinabulin**	Halimide (Fungus)	Diketopiperazine	Microtubules	NSCLC ^e^; CIN ^f^
**Salinosporamide A** **Marizomib**	Salinosporamide A (Bacterium)	γ-lactam-β-lactone	20S proteasome	Newly diagnosed glioblastoma
**Polatuzumab vedotin** **DCDS-4501A**	Dolastatin 10	ADC (MMAE)	CD79b and microtubules	DLBCL ^g^
**Depatuxizumab vedotin** **ABT-414**	Dolastatin 10	ADC (MMAF ^h^)	EGFR and microtubules	Newly diagnosed glioblastoma
**PHASE 2**				
**PM060184** **Plocabulin**	PM060184 (Sponge)	Polyketide	Microtubules	Advanced colorectal cancer
**Enfortumab vedotin** **ASG-22ME**	Dolastatin 10	ADC (MMAE)	Nectin-4 and microtubules	Carcinoma, transitional cell; urinary bladder, urologic, renal pelvis, ureteral and urethral neoplasms; urothelial cancer;
**Glembatumumab vedotin** **CDX-011**	Dolastatin 10	ADC (MMAE)	gpNMB and microtubules	Metastatic gpNMB over-expressing triple negative breast cancer; recurrent osteosarcoma; recurrent uveal melanoma; stage IV uveal melanoma AJCC v7; melanoma; squamous cell carcinoma of the lung
**AGS-16C3F**	Dolastatin 10	ADC (MMAF)	ENPP3 and microtubules	Metastatic renal cell carcinoma
**GSK2857916**	Dolastatin 10	ADC (MMAF)	BCMA	Multiple myeloma
**Tisotumab vedotin** **(HuMax^®^-TF-ADC)**	Dolastatin 10	ADC (MMAE)	Tissue factor and microtubules	NSCLC; Ovary, cervical, endometrium, bladder, prostate and esophagus cancer; squamous cell carcinoma of the head and neck
**Ladiratuzumab vedotin** **SGN-LIV1A**	Dolastatin 10	ADC (MMAE)	LIV-1 and microtubules	Breast cancer
**Telisotuzumab vedotin** **ABBV-399**	Dolastatin 10	ADC (MMAE)	c-Met	Recurrent and stage IV squamous cell lung carcinoma; NSCLC
**PHASE 1**				
**ABBV-085**	Dolastatin 10	ADC (MMAE)	LRRC15	Advanced solid tumors; undifferentiated pleomorphic sarcoma; squamous cell carcinoma of the head and neck; breast carcinoma
**AGS-67E**	Dolastatin 10	ADC (MMAE)	CD37 and microtubules	Refractory/relapsed lymphoid malignancy
**ASG-15ME**	Dolastatin 10	ADC (MMAE)	SLITRK6 and microtubules	Metastatic urothelial cancer
**PF-06647020**	Dolastatin 10	ADC (PF-06380101)	PTK7 and microtubules	Advanced solid tumors; triple negative and metastatic breast cancer

^1^ Based on the latest stage of clinical development registered on the US clinical trials database (recruiting; active, not recruiting; not yet recruiting; and enrolling by invitation) and/or EU clinical trials (ongoing). ^a^ Antibody–drug conjugate; ^b^ monomethyl auristatin E; ^c^ systemic anaplastic large-cell lymphoma; ^d^ small-cell lung cancer; ^e^ non-small-cell lung cancer; ^f^ chemotherapy-induced neutropenia; ^g^ diffuse large B-cell lymphoma; ^h^ monomethyl auristatin F.
